# An Epidemic of Jaundice with Some Unusual and Remarkable Symptoms

**Published:** 1880-04

**Authors:** R. J. Nunn

**Affiliations:** Savannah, Ga.


### AN EPIDEMIC OF JAUNDICE WITH SOME UNUSUAL AND REMARKABLE SYMPTOMS.

BY R. J. NUNN, M.D., SAVANNAH, GA.

During the months of January and February of this year, 1880, a slight epidemic of jaundice prevailed in this city possessing some points of interest which it is well to record without, however, going into an elaborate discussion of the subject.

The attack commenced by a feeling of malaise, generally followed by nausea and vomiting; sometimes this ushered in a twenty-four hours fever of moderate severity. In the severer cases of this type, vomiting continued for a day. Frontal pain and headache were all present, and early in the progress of the case the conjunctiva was noticeably yellow. Constipation was usual, but sometimes there was diarrhœa. The color of the dejection did not indicate an absence of bile. The urine was scanty and high colored or sometimes even altogether suppressed for as much as forty-eight hours. The temperature fell as much as two (2) degrees below the normal standard. The pulse generally sank to sixty per minute and, in extraordinary cases, sometimes much lower, in one case falling to forty-eight. At some period of the attack, there was generally great pain and tenderness over the anterior border of the liver, and in about a dozen of the cases reported, this was a prominent, annoying and most persistent symptom, and these particular cases were much prostrated and made a slow convalescence. Respiration was unaffected unless in cases where it was quickened by pain, and in the majority of cases, the headache disappeared after the first few days of the attack. There was loss of appetite; tongue coated, principally toward the edges, with a red center and tip; thirst was not unusually great; rest was disturbed.

The duration of the active period of the attack rarely exceeded two days. The disease was self-limited, requiring no medication unless for complications. It was also monoparoxysmal; the skin was cool to the touch; there was great physical and nervous depression with much anxiety of expression; jaundice was most marked, but not very persistent, passing off in a few days. Recovery was tedious in proportion to the gravity of the symptoms; pains and weakness in the legs, with a tendency to cramps, persisting for some time; the appetite and the powers of assimilation appeared to be for a long time impaired.

About eighty cases of this disease were reported to the Georgia Medical Society, of which about a dozen were of the severer type referred to in the body of this paper.

The peculiar points of interest to which I would wish to direct attention, are the low temperature, depressed pulse, the monoparoxysmal, self-limited character of the disease, the suppression of urine and the striking similarity in these and other respects, which exists between these cases and the second stage or stages of depression of yellow fever. No fatal cases have come to my knowledge.

It might be well to mention that the past winter has been unusually warm, the thermometer seldom falling below summer heat.

